# Selective Membrane Protein Enrichment Enables Defined Biomimetic Nanoparticles for Endothelial Targeting

**DOI:** 10.1002/smll.202513548

**Published:** 2026-01-12

**Authors:** Sivan Arber Raviv, Rawan Mhajne, Maayan Ben‐Eliezer, Tamar Gross Lev, Assaf Zinger

**Affiliations:** ^1^ Bioinspired Nano Engineering and Translational Therapeutics Lab Department of Chemical Engineering Technion−Israel Institute of Technology Haifa Israel; ^2^ Russell‐Berrie Nanotechnology Institute Technion – Israel Institute of Technology Haifa Israel; ^3^ Cardiovascular Sciences Department Houston Methodist Academic Institute Houston Texas USA; ^4^ Neurosurgery Department Houston Methodist Academic Institute Houston Texas USA; ^5^ Resnick Sustainability Center of Catalysis Technion−Israel Institute of Technology Haifa Israel; ^6^ Bruce and Ruth Rappaport Cancer Research Center Technion−Israel Institute of Technology Haifa Israel

**Keywords:** bioengineering, biomimetic nanoparticles, drug delivery, inflammation, membrane proteins, translational research

## Abstract

Nanoparticles offer a promising strategy for targeted drug delivery while reducing off‐target toxicity. Biomimetic nanoparticles, which integrate native cell components, enhance biological compatibility but often suffer from poorly defined protein compositions that hinder reproducibility and clinical translation. Here, we present a next‐generation biomimetic approach to engineer Particular Nanoparticles (PNPs)‐ formulation enriched with specific, functionally relevant membrane proteins for precise control and tunability. We incorporated leukocyte adhesion proteins (including CD18, CD11a, and CD11b) into the nanoparticle membrane to enhance targeting of inflamed sites. Using a 2D microfluidic model that mimics human blood vessels, adhesion‐enriched PNPs demonstrated significantly improved endothelial interactions and greater accumulation under flow at inflamed endothelium compared to conventional Leukosomes. This protein‐defined biomimetic nanoparticle platform offers enhanced targeting efficiency, improved reproducibility, and translational potential for inflammation‐targeted therapies.

## Graphical abstract

Particular nanoparticles (PNPs), engineered with defined enrichment of leukocyte adhesion membrane proteins, demonstrate superior targeting of inflamed sites in a microfluidic inflamed vessel model under flow conditions. This selective membrane protein enrichment enhances targeting efficiency while improving nanoparticle production reproducibility and translational potential for inflammation‐targeted drug delivery.

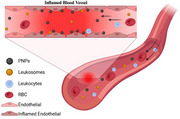

## 1 Introduction

Lipid‐based nanoparticles (NPs) have been widely used in medicine since the 1960s to enhance drug efficacy, protect therapeutic cargo, and minimize systemic side effects [1, 2, 3, 4, 5]. Their clinical impact is well established, with several liposomal formulations demonstrating improved pharmacokinetics and reduced toxicity in patients [6]. Despite extensive optimization, clinical translation remains limited by rapid immune clearance and insufficient accumulation in target tissues [7, 8]. Current strategies, including polyethylene glycol (PEG) modification to prolong circulation and conjugation of targeting ligands, face inherent limitations [4, 9, 10, 11]. PEGylation can trigger adverse immune responses upon repeated dosing, while ligand‐based targeting strategies are constrained by complex production and poor formulation control [4, 12]. Moreover, despite decades of research, systemically administered nanoparticles still exhibit poor targeting efficiency, limiting their ability to selectively reach diseased tissues. For example, in tumor therapy, less than 1.5% of injected nanoparticles typically accumulate in solid tumors, highlighting the urgent need for delivery systems that can navigate biological barriers and achieve selective accumulation at disease sites [13].

Biomimetic nanoparticles (BNPs) have emerged as a promising class of nanocarriers by leveraging native cell‐derived components to mimic physiological cell–cell communication to improve biological interfacing [14, 15, 16, 17, 18]. Conventional BNP fabrication relies on either (i) cell membrane fragments, such as generating nanoghosts [19, 20], or (ii) incorporation of membrane proteins (MPs) into synthetic lipid scaffolds [14, 21]. For example, Leukosomes, which integrate MPs derived from mouse macrophages, demonstrated that increasing the protein‐to‐lipid ratio (P:L ratio) enhanced association with triple‐negative breast cancer and lipopolysaccharide (LPS)‐induced local inflammation in mouse models, with 1:20 P:L ratio showing the highest accumulation compared to 1:40 and 1:100 [14].

While effective, these approaches rely on the nonspecific incorporation of whole MPs extracts. The lack of control over MPs identity results in poorly defined formulations containing redundant or counterproductive proteins, and uncontrolled stoichiometry has limited mechanistic understanding of nanoparticle–cell interactions and hindered clinical translation. Addressing this gap requires a strategy to selectively enrich and integrate functional MPs that mediate biologically meaningful adhesion and signaling [22].

Herein, we hypothesized that selectively enriching monocyte‐derived adhesion MPs, particularly selectins and integrins, would enhance nanoparticle targeting and accumulation at inflamed endothelium by replicating the multivalent, shear‐dependent interactions that govern leukocyte trafficking [23]. Initially, development focused on directly integrating specific MPs, such as CD18, CD11b, and L‐selectin, to mimic leukocyte behavior. However, isolating individual MPs is inherently challenging, costly, and yields low quantities, making direct incorporation impractical for scalable and clinically compliant formulations [24, 25, 26]. Thus, there is an unmet need for strategies that enable the selective incorporation of functionally relevant MPs to generate defined and reproducible BNPs.

To address this, we introduce Particular Nanoparticles (PNPs), a new class of biomimetic nanocarriers that selectively integrate functionally relevant MPs according to their biological role. Unlike Leukosomes, which incorporate whole protein extracts, PNPs selectively incorporate enriched, functionally relevant MPs, yielding defined and tunable formulations. This strategy is designed to enhance targeting, improve reproducibility, and provide a scalable path toward clinical translation in targeted drug delivery.

As a proof of concept, we focused on targeting inflamed vascular endothelium, a hallmark of numerous pathologies including infection, cancer, stroke, cardiovascular disorders, and chronic inflammatory diseases [11, 27, 28]. We aimed to mimic monocytes, a subset of leukocytes that circulate in the bloodstream and migrate toward inflamed tissues through specific adhesion and signaling mechanisms. Upon activation, monocytes tether, roll, and adhere to endothelial cells via adhesion MPs before transmigrating into inflamed tissues [23]. Monocyte rolling and firm adhesion are favored under physiological shear stress, as selectin‐mediated interactions require flow to stabilize transient bonds between monocyte and endothelial cells [29, 30, 31]. Under static conditions, these interactions are markedly weaker, leading to minimal accumulation [31]. Endothelial cells upregulate ligands such as E‐selectin, P‐selectin, and ICAM‐1 during inflammation, which mediate these adhesion processes [32].

Guided by this biological framework, we enriched monocyte‐derived adhesion‐related MPs using size‐exclusion chromatography (SEC) and incorporated them into lipid NPs to generate PNPs. To ensure translational relevance, all experiments were conducted using human monocyte and endothelial cell lines, providing a clinically meaningful in vitro model for evaluating endothelial targeting under both static and flow conditions. This approach establishes a foundation for controlled, function‐driven, and clinically translatable biomimetic nanoparticle design.

Beyond inflammation, this modular enrichment strategy could be adapted to engineer NPs displaying alternative cell‐type–specific proteins, enabling precision delivery across a wide range of pathological contexts. Using a relatively common method such as SEC, we enriched specific MPs‐ an approach that can be extended to a variety of desired membrane components to overcome the limitations of single‐protein functionalization. By decoupling biological functionality from undefined membrane extracts, PNPs establish a new framework for reproducible, mechanism‐driven design of clinically translatable biomimetic nanomedicines.

## 2 Results and Discussion

**FIGURE 1:**
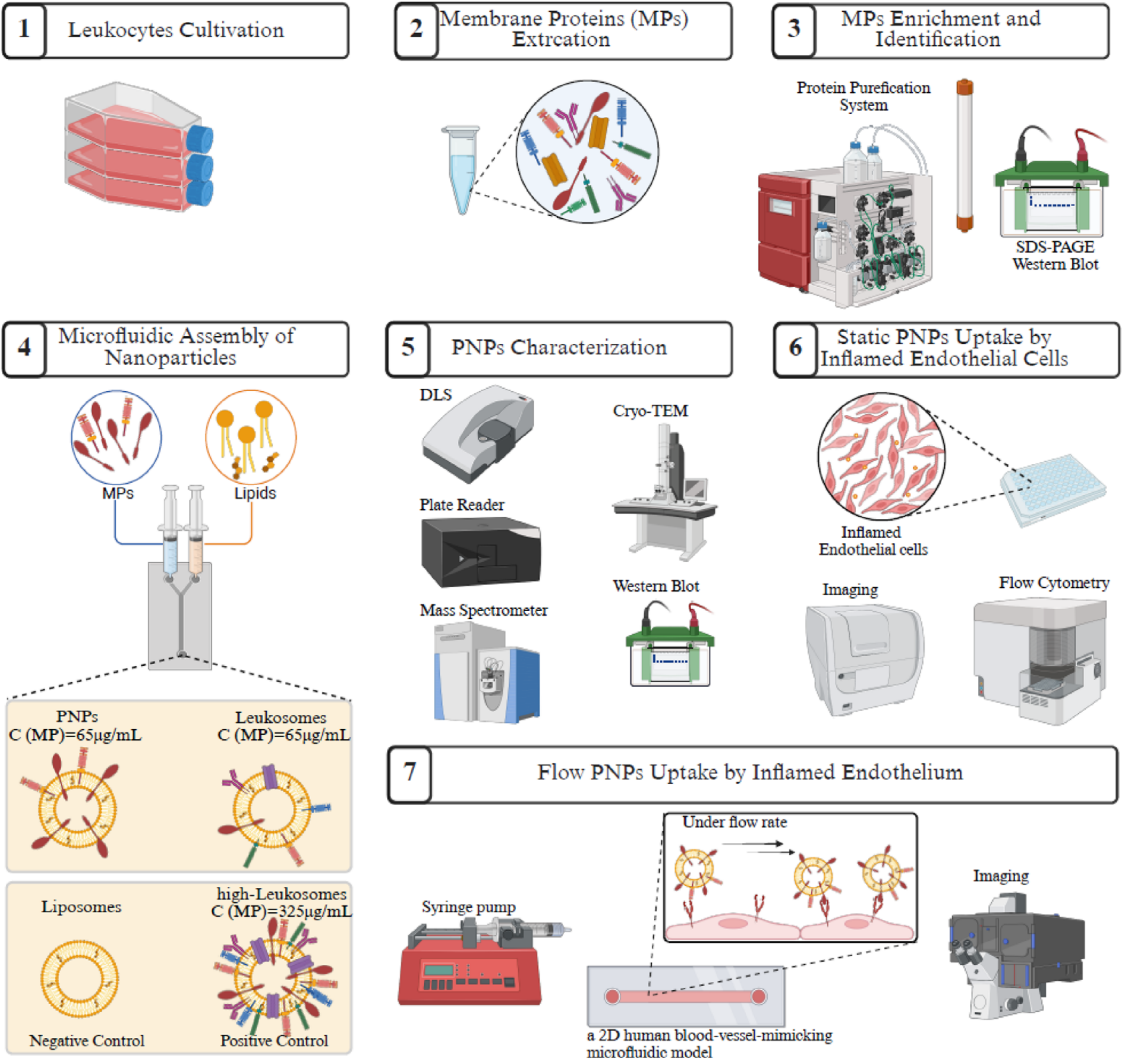
Schematic illustration of the selective membrane protein (MPs) enrichment and PNPs assembly for enhanced biological targeting and formulation precision. (1) Human leukocyte (THP‐1) cells are cultured, followed by (2) membrane protein extraction. (3) Adhesion‐related MPs are enriched using size exclusion chromatography (SEC) and validated by SDS–PAGE and western blot. (4) Selected MPs are incorporated during PNP assembly to yield a protein‐defined biomimetic formulation. (5) PNPs are characterized for physicochemical stability and biomimetic features. (6) Cellular uptake is evaluated in lipopolysaccharide (LPS)‐inflamed human umbilical vein endothelial cells (HUVECs) under static conditions. (7) Cellular uptake is evaluated under dynamic (shear flow) conditions. Illustration created with https://www.biorender.com/BioRender.com.

To address the limitations of current biomimetic nanocarriers that rely on undefined mixtures of MPs^22^, this study introduces a next‐generation biomimetic platform for engineering PNPs, liposomes functionalized with selectively enriched leukocyte‐derived MPs. Using a controlled microfluidic assembly process, we integrate adhesion‐related MPs to achieve protein‐defined, tunable formulations with improved biological specificity. The resulting PNPs were systematically characterized and evaluated for their accumulation on inflamed endothelial cells (Figure 1). A detailed summary of the nanoparticle compositions and control formulations is provided in Table 1.

**TABLE 1:** Summary of nanoparticle formulations highlighting Membrane Protein (MP) source, enrichment strategy, and composition. P:L ratio stands for protein to lipid ratio.

	Formulation	MPs Source	Enrichment Strategy	P:L ratio (MP concentration)	Control
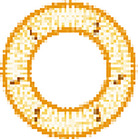	Liposomes	None	None	None	Negative control
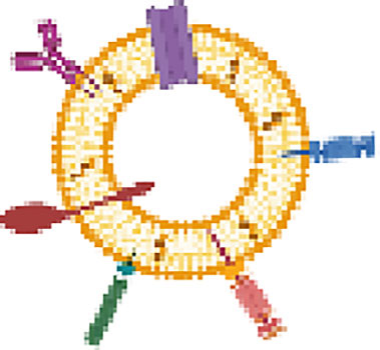	Leukosomes	THP‐1 Leukocytes	Whole MPs extracted	1:100 (65 µg mL^−1^)	
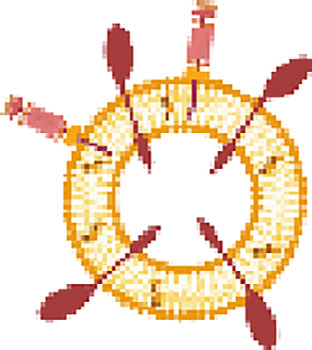	PNPs	THP‐1 Leukocytes	Enriched adhesion MPs	1:100 (65 µg mL^−1^)	
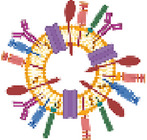	High‐Leukosomes	THP‐1 Leukocytes	Whole MPs extracted	1:20 (325 µg mL^−1^)	Positive control

### 2.1 Selective Enrichment of Adhesion MPs and Optimization of PNPs Assembly Conditions

**FIGURE 2:**
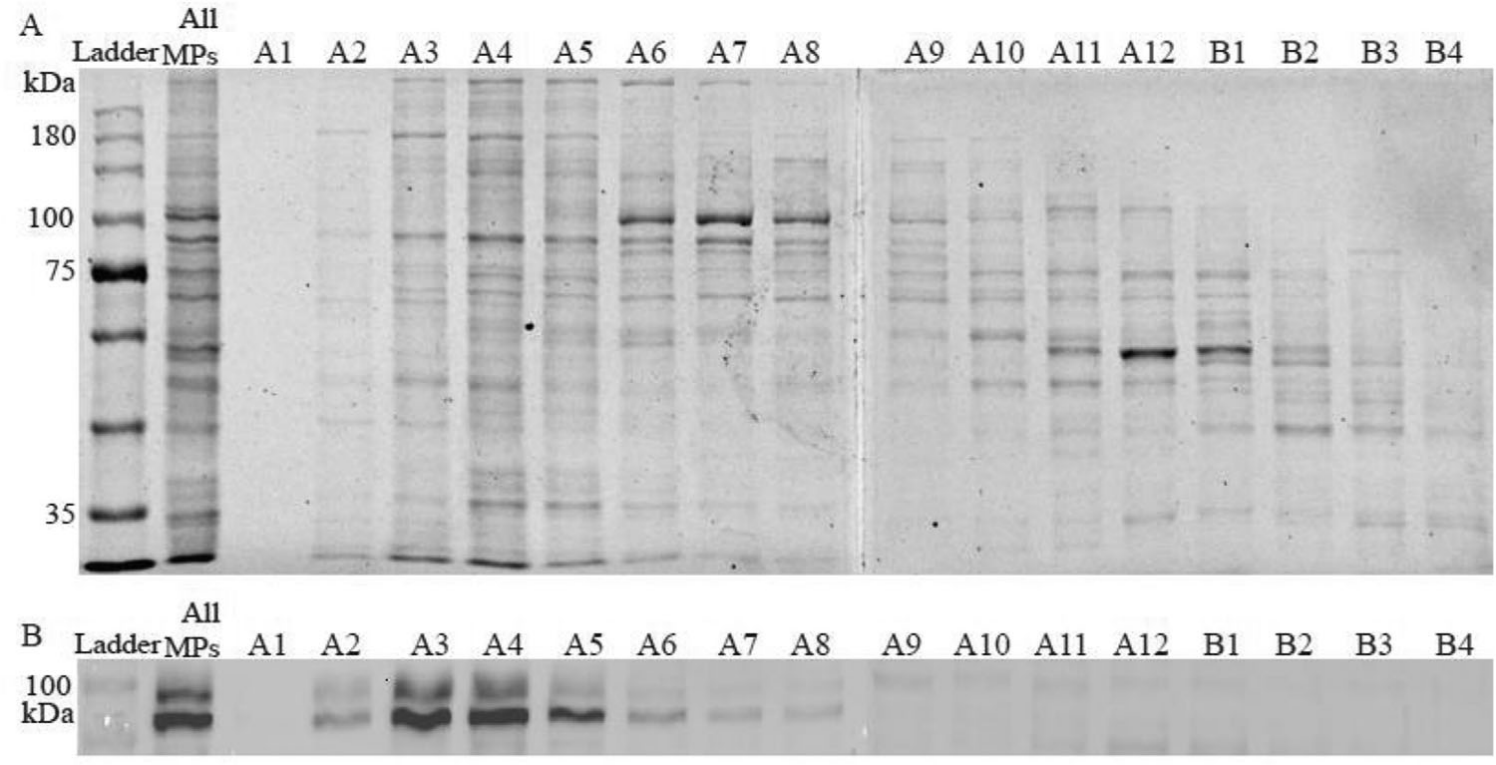
Size‐Exclusion Chromatography (SEC) Enables Selective Enrichment of Adhesion MPs for Defined PNP Formulation. (A) SDS–PAGE of SEC fractions obtained using a Superdex 200 Increase column, confirming molecular‐weight–based protein separation. (B) Western blot analysis (exposure time: 0.075 s) showing CD18 enrichment in fractions A3–A5. Collectively, these data demonstrate that using fractions A3–A5 for PNPs synthesis enhances incorporation of CD18 and other high–molecular‐weight adhesion‐related MPs, yielding a defined biomimetic formulation.

To selectively enrich adhesion MPs, human THP‐1 monocytes were cultured and processed using the NEXT protocol [33]. This method yielded an average of 18 ± 7 µg mL^−1^ of MPs per million cells. Because many adhesion‐related MPs exceed ∼85 kDa (Table 2), size exclusion chromatography (SEC) was employed for size‐based enrichment (Figure S1A). SDS–PAGE analysis confirmed efficient molecular‐weight separation (Figure 2A), and western blot detection of CD18‐ a representative adhesion protein near the target cutoff‐ verified enrichment of high‐molecular‐weight components alongside depletion of smaller proteins. Fractions A3–A5, which showed clear CD18 enrichment, were selected for PNP synthesis (Figures 2B; S1B).

**TABLE 2:** Summary of adhesion‐related MPs identified from THP‐1 monocytes, highlighting proteins involved in cell adhesion and immune recognition. Proteins with molecular weights ≥85 kDa were prioritized to enrich key adhesion components. CD18 (β_2_ integrin) served as a representative marker for successful enrichment.

Protein Name	Gene Name	Adhesion Role [23, 34]	Ligand on Endothelium	Mw (kDa) [34]
CD11a (LFA‐1 α subunit)	ITGAL	Firm adhesion, Emigration	ICAM‐1	129
CD11b (Mac‐1 α subunit)	ITGAM	Firm adhesion, Emigration	ICAM‐1, fibrinogen	127
CD11c	ITGAX	Adhesion	ICAM‐1, fibrinogen	128
ESL‐1	GLG1	Adhesion	E‐selectin	135
Integrin α4	ITGA4	Firm adhesion	VCAM‐1	115
CD31	PECAM1	Emigration	PECAM1 (homophilic)	83
CD44	CD44	Adhesion, migration	Hyaluronan	82
CD18 (β2 integrin)	ITGB2	Mediates firm adhesion	ICAM‐1, ICAM‐2	85
CD29 (Integrin β1)	ITGB1	Firm adhesion	VCAM‐1	88
CD97	CD97	Migration	CD55, chondroitin sulphate	92
SELPLG (PSGL‐1)	SELPLG	Capture, rolling	P‐selectin, E‐selectin	43
CD62L (L‐selectin)	SELL	Capture, rolling	P‐selectin, E‐selectin	43

Initial PNPs assembly experiments revealed the critical influence of detergent concentration on the NPs stability. When SEC was performed using a buffer containing 0.5% (v/v) Triton X‐100 followed by MP concentration, the resulting PNPs exhibited poor colloidal stability and substantial particle enlargement (Table S1). Elevated detergent levels above 0.1% (v/v) interfered with phospholipid self‐assembly, leading to vesicle instability and particle expansion to ∼380 nm. Additional tests across varying total flow rates (TFR) further supported this observation.

**FIGURE 3:**
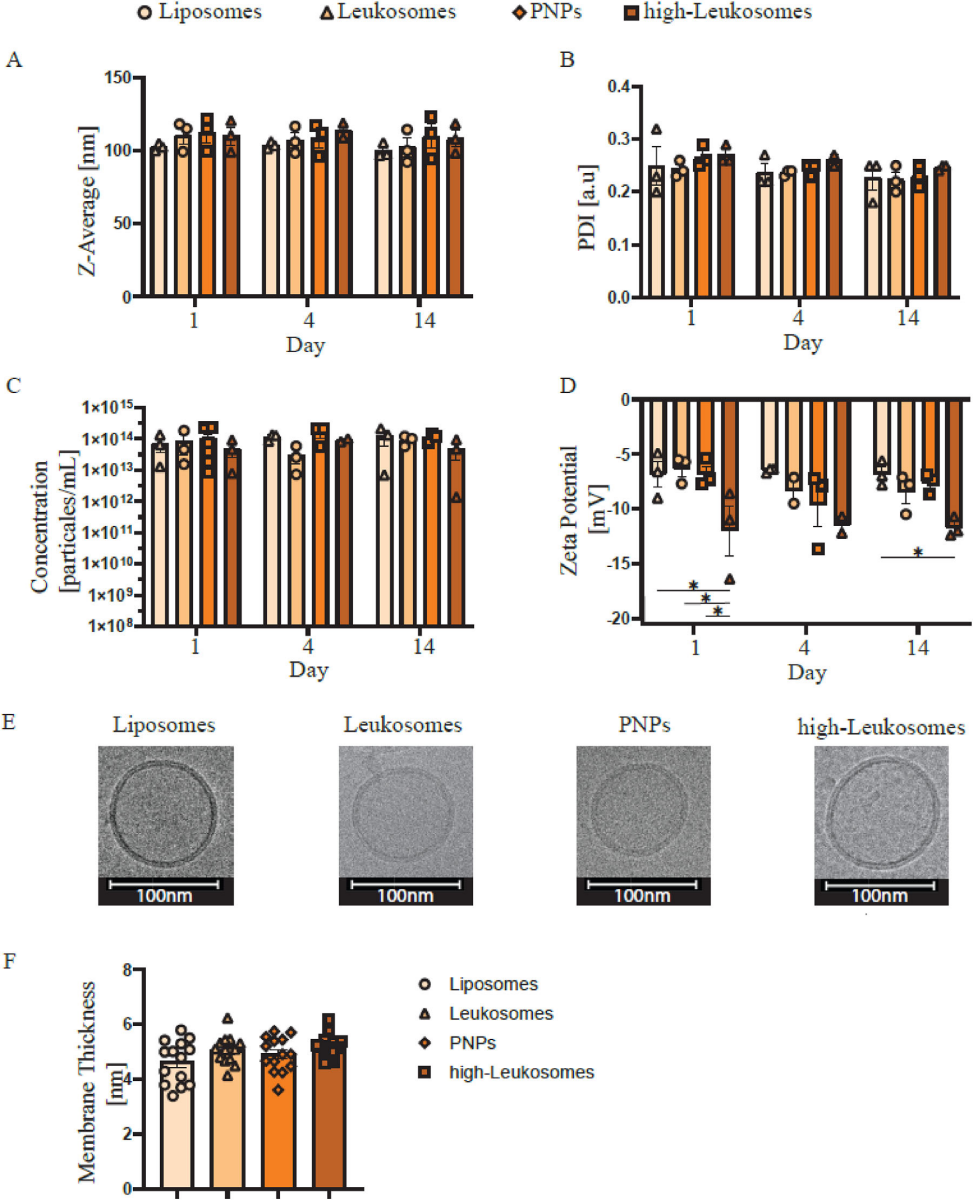
Controlled Microfluidic Assembly Yields Reproducible and Stable PNPs. PNPs were characterized for (A) hydrodynamic diameter (Z‐average), (B) polydispersity index (PDI), (C) particle concentration, and (D) zeta potential over 14 days at 4°C using dynamic light scattering (DLS), confirming high reproducibility and long‐term stability. (E) Cryo‐transmission electron microscopy (cryo‐TEM) images verified vesicular morphology and uniform size distribution across formulations. (F) Quantitative analysis of membrane thickness from cryo‐TEM images showed no significant differences among formulations (*n* = 14). For (A‐D), data are presented as mean ± SEM (*n* = 3–6 per formulation) and statistical analyses were performed using two‐way ANOVA followed by Tukey's multiple comparisons test (^*^ *p* < 0.05). For (F), data are presented as mean ± SEM (*n* = 14 per formulation) and were analyzed using one‐way ANOVA followed by Tukey’s multiple‐comparisons test (*P* < 0.05).

In contrast, performing SEC with a detergent‐free buffer enabled proper phospholipid self‐assembly, yielding stable PNPs with an average hydrodynamic diameter of ∼100 nm (Figure 3A), suitable for subsequent functional evaluation. Collectively, these findings demonstrate that SEC effectively enable size‐based enrichment of high‐molecular‐weight adhesion MPs, facilitating controlled and reproducible PNP formulation.

### 2.2 Optimized Microfluidic Assembly and NPs Physicochemical Characterization

Before formulation optimization, we defined the target physicochemical profile for NPs: a mean diameter of ∼100 nm, polydispersity index (PDI) ≤ 0.25, negative zeta potential, and stability for at least 14 days to ensure suitability for biological applications.

Controlled microfluidic synthesis using the Helix system enabled reproducible production of stable NPs (Figure 3A–D). Both PNPs and Leukosomes were prepared with a total MPs concentration of 65 µg mL^−1^ (protein‐to‐lipid ratio, P:L = 1:100). Leukosomes contained the full, non‐selective MP extract from THP‐1 cells, while PNPs incorporated only the adhesion enriched‐ MP fraction. A high‐Leukosomes formulation prepared with a fivefold higher MP concentration (325 µg mL^−1^, P:L = 1:20), served as a positive control as previously described [14]. Liposomes were used as negative control.

The total flow rate (TFR) during microfluidic assembly was optimized to achieve the desired particle size of ∼100 nm. Variations in MPs concentration and solution volume altered residual Triton X‐100 concentrations, requiring TFR adjustments to maintain consistent self‐assembly conditions. For high‐Leukosomes, a TFR of 2.4 mL min^−1^ was optimal, whereas 2.2 mL min^−1^ sufficed for Leukosomes, PNPs, and liposomes. Mechanistically, higher TFR enhances mixing and accelerates lipid nucleation, producing smaller, more uniform vesicles, while insufficient mixing yields larger, heterogeneous aggregates.

Following optimization, all NPs formulations exhibited an average hydrodynamic diameter of 110 ± 10 nm (Figure 3A), and a polydispersity index (PDI) of approximately 0.25 ± 0.02 (Figure 3B), indicating uniform and stable NPs populations. NPs concentrations were consistent across all groups, averaging (1.00 ± 0.04) × 10^1^⁴ particles mL^−1^, with no significant differences observed (Figure 3C). Zeta potential (ZP) analysis revealed that high‐Leukosomes exhibited a more negative surface charge (−9 ± 2 mV) than Liposomes, Leukosomes, and PNPs‐1.9‐, 1.7‐, and 1.6‐fold more negative, respectively‐likely reflecting their elevated MPs content (Figure 3D). Cryo‐transmission electron microscopy (cryo‐TEM) confirmed vesicular morphology, expected bilayer structure, and comparable sizes across formulations (Figure 3E–F).

Finally, physicochemical stability assessments at days 1, 4, and 14 showed no significant changes in size, PDI, NPs concentration, or ZP, confirming that all formulations remained stable for at least 14 days when stored at 4°C.

### 2.3 Protein Composition and Biomimetic Characterization of Nanoparticles

**FIGURE 4:**
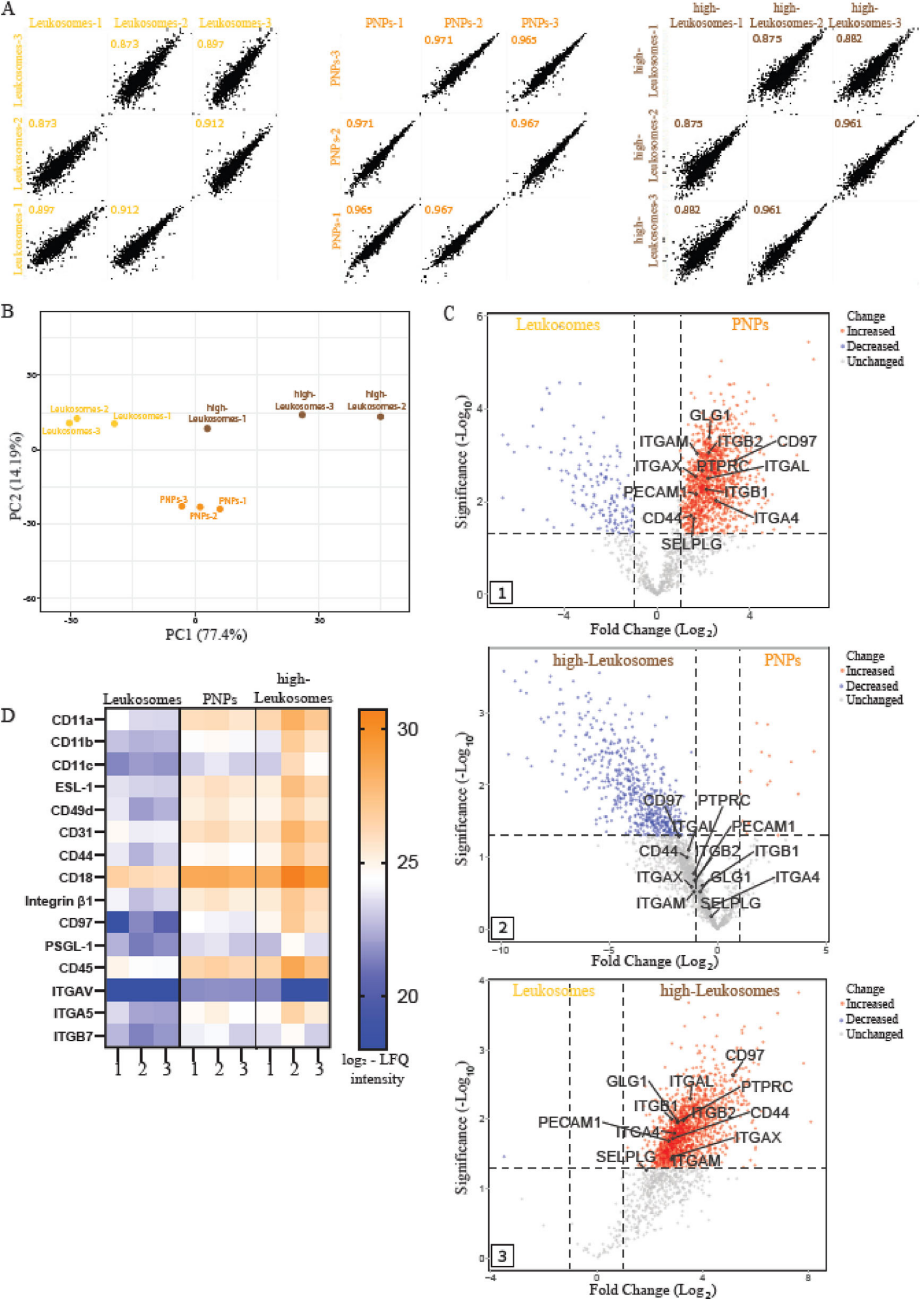
Proteomic Analysis Reveals High Reproducibility and Selective Adhesion Protein Enrichment in PNPs. (A) Multi‐scatter plots of protein intensity values (*n* = 3) show the highest intra‐group reproducibility for PNPs compared to Leukosomes and high‐Leukosomes. (B) Principal component analysis (PCA) demonstrates tight clustering of PNPs and clear separation between formulations (PC1 = 77%, PC2 = 14%). (C) Volcano plots show differential protein abundance between (i) PNPs vs Leukosomes, (ii) PNPs vs high‐Leukosomes, and (iii) Leukosomes vs high‐Leukosomes (|log_2_ Fold change| ≥ 1, *p* ≤ 0.05, FDR = 0.05). Red = enriched, blue = depleted, grey = no change. (D) Heatmap of adhesion‐related MPs shows selective enrichment in PNPs.

To assess MPs enrichment and formulation reproducibility, the protein composition of each NPs formulation was analyzed by mass spectrometry across three independent batches (Figure 4).

Proteomic analysis revealed that the PNPs formulation exhibited the most consistent and well‐defined protein profile compared to Leukosomes and high‐Leukosomes formulations (Figure 4A,B). Multi‐scatter plots of protein intensity values demonstrated high intra‐group reproducibility for PNPs, with Pearson correlation coefficients (R) of 0.968 ± 0.003, 0.89 ± 0.02, and 0.91 ± 0.05 for PNPs, Leukosomes, and high‐Leukosomes, respectively (Figure 4A, *n* = 3).

Principal component analysis (PCA) further confirmed these findings, showing tight clustering of PNPs replicates compared to the broader distribution of Leukosomes and high‐Leukosomes (Figure 4B). The distinct separation between formulations indicates both reproducibility and compositional specificity. PC1 and PC2 accounted for 77% and 14% of the total variance, respectively. The reduced variability within the PNP group likely reflects the selective incorporation of adhesion‐related MPs, resulting in a more defined and stable formulation.

Comparative analysis of MP components between formulations revealed a clear enrichment of adhesion‐related proteins in PNPs. Volcano plot analyses highlighted significant compositional differences across NPs types. When comparing PNPs to Leukosomes, PNPs showed marked enrichment of adhesion proteins‐including CD11a, CD11b, and CD18, which were increased by 4.6−, 3.3−, and 4.7‐fold, respectively‐accompanied by a depletion of unrelated proteins (Figure 4C1). Comparison of PNPs to high‐Leukosomes revealed similar adhesion MP levels but a reduced abundance of non‐adhesion proteins, confirming that PNPs maintained selective enrichment despite a fivefold lower total MP input (Figure 4C2). As expected, Leukosomes and high‐Leukosomes displayed comparable protein profiles differing primarily in total MP content (Figure 4C3).

Heatmap analysis of adhesion‐related MPs further emphasized their selective enrichment in PNPs relative to Leukosomes (Figure 4D; *n* = 3). Remarkably, PNPs exhibited an adhesion protein profile comparable to that of high‐Leukosomes, despite lower total MP content.

Collectively, these results demonstrate that SEC‐based enrichment effectively concentrates biologically relevant adhesion proteins, yielding a reproducible, compositionally defined, and functionally enriched biomimetic nanoparticle formulation.

### 2.4 Evaluation of PNPs for Enhanced Endothelial Targeting

Targeting inflamed endothelium is a central therapeutic strategy for many inflammatory and autoimmune diseases [35]. To this end, we developed BNPs designed to engage inflamed endothelium under physiological flow conditions. To enhance clinical relevance and reduce animal use, we employed a 2D human blood‐vessel–mimicking microfluidic model rather than murine systems. Human THP‐1 monocytes and human umbilical vein endothelial cells (HUVECs) were selected to replicate monocyte circulation and vascular inflammation. NPs accumulation was assessed under both static and flow conditions to evaluate targeting performance.

**FIGURE 5:**
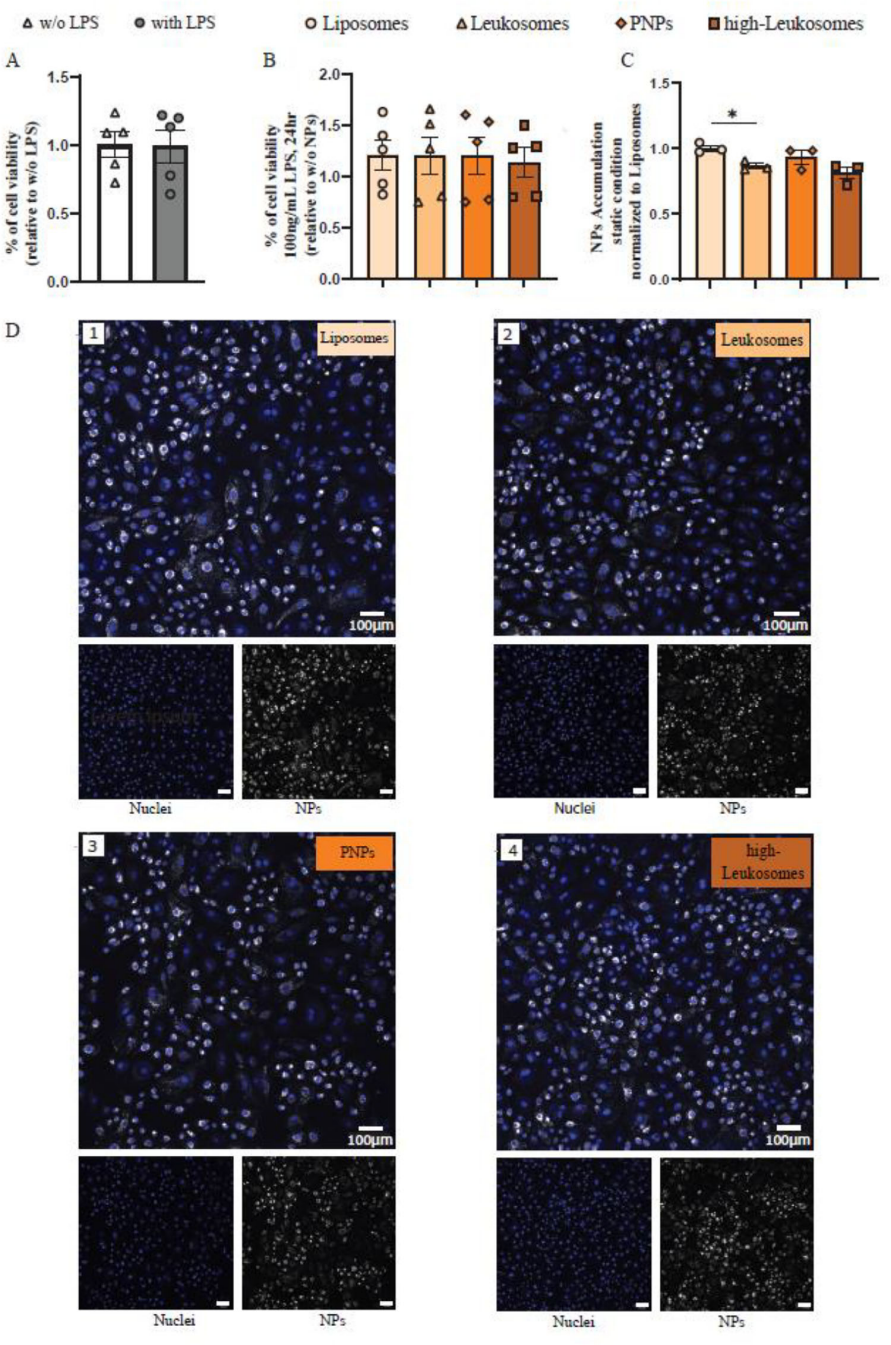
PNPs are Cytocompatible and Do Not Exhibit Enhanced Accumulation with Inflamed Endothelial Cells Under Static Conditions. (A) Cytotoxicity of LPS (100 ng mL^−1^, 24 h) in HUVECs assessed by MTT assay (*n* =5). (B) Cytocompatibility of biomimetic nanoparticles (BNPs) in LPS‐pretreated HUVECs following 1 h incubation with 0.1 mm BNPs (*n* =5). (C) Quantification of PNPs association with LPS‐stimulated HUVECs after 1 h incubation with 0.1 mm NPs. Fluorescence intensity per cell was normalized to liposome‐treated controls and relative NPs fluorescence. Data represent mean ± SEM (*n* = 3). Statistical analysis was performed using Welch's one‐way ANOVA followed by Games–Howell post hoc test. (D) Representative Z‐stack spinning disk confocal images (10X magnification) showing rhodamine‐labeled NPs (gray) association to inflamed HUVECs; nuclei stained with Hoechst (blue). Scale bar = 100 µm.

Before conducting accumulation studies, we assessed the cytocompatibility of the experimental conditions. Treatment of HUVECs with lipopolysaccharide (LPS, 100 ng mL^−1^, 24 h) did not reduce cell viability compared to untreated controls (Figure 5A). Similarly, exposure of inflamed or non‐inflamed HUVECs to 0.1 mM NPs for 1 h under static conditions caused no detectable cytotoxicity (Figures 5B; S2).

Under static conditions, fluorescently labeled NPs were incubated (0.1 mm, 1 h) with confluent HUVEC monolayers (LPS 100 ng mL^−1^, 24 h). As expected, spinning disk confocal (SDC) imaging revealed that monocyte‐derived BNPs did not show increased accumulation under static conditions compared to liposomes [31, 36, 37]. Moreover, PNPs, Leukosomes, and high‐Leukosomes tended to accumulate less than liposomes (*n* = 3) (Figure 5C,D).

Next, PNPs accumulation was evaluated under physiologically relevant flow conditions to assess shear‐dependent adhesion [29, 31]. Fluorescently labeled NPs (0.05 mm) were perfused over confluent LPS‐stimulated HUVEC monolayers (100 ng mL^−1^, 24 h) within µ‐slide microfluidic channels at a flow rate of 0.57 ± 0.02 mL min^−1^ for 12 min.

**FIGURE 6:**
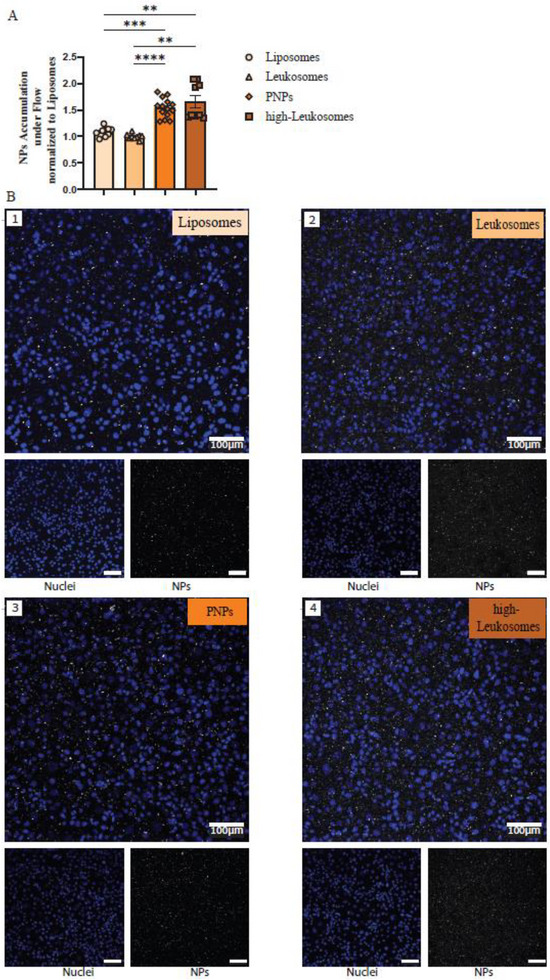
Selective Adhesion Protein Enrichment Enables Shear‐Dependent Endothelial Targeting of PNPs. (A) Quantification of PNPs association with LPS‐stimulated HUVECs after 12 min of flow (0.05 mM BNPs; 0.57 ± 0.02 mL min^−1^; 0.5 dyn cm^−^^2^). Fluorescence intensity per cell was normalized to liposome‐treated controls and to the relative fluorescence of free BNPs. (B) Representative Z‐stack SDC images (20X magnification) showing uptake of rhodamine‐labeled BNPs (gray) by inflamed HUVECs; nuclei stained with Hoechst (blue). Scale bar = 100 µm. Data are presented as mean ± SEM (*n* = 8–12; 4 frames per slide, 2–3 slides). Statistical analysis was performed using one‐way ANOVA followed by Tukey's multiple comparison test.

Quantitative image analysis revealed a significant increase in PNPs accumulation compared to Leukosomes (1.54 vs. 1.09; *p* < 0.0001) (Figure 6A), confirming enhanced adhesion mediated by selective MPs enrichment. We hypothesize that this effect arises from PSGL‐1, a constitutively active selectin ligand that forms shear‐dependent catch bonds with P‐ and E‐selectins on endothelial cells, allowing transient contacts to stabilize under flow and promoting firm adhesion [29]. Notably, PNPs achieved adhesion comparable to high‐Leukosomes, despite the latter containing approximately fivefold higher total MPs content. Representative SDC illustrate these differences (Figure 6B).

## 3 Conclusions

Selective enrichment of membrane proteins via size‐exclusion chromatography (SEC) enables the controlled and reproducible formulation of PNPs with defined adhesion‐molecule enrichment. Incorporating CD18‐enriched membrane fractions from THP‐1 monocytes markedly enhanced adhesion‐protein content while minimizing compositional variability, resulting in improved batch‐to‐batch consistency. Microfluidic synthesis further ensured uniform particle size distribution, optimal physicochemical stability, and consistent production of nanoparticles that remained stable for at least 14 days under storage conditions.

Biological characterization demonstrated that while PNPs showed no preferential interaction with endothelial cells under static conditions, consistent with the shear‐dependent nature of leukocyte adhesion, PNPs exhibited markedly enhanced accumulation under flow [29, 31]. Importantly, PNPs adhered more strongly to inflamed endothelial cells than standard Leukosomes formulated with equivalent membrane‐protein content, and achieved comparable adhesion to high‐Leukosomes containing approximately fivefold greater total membrane protein levels. This finding underscores the critical role of selective protein enrichment over bulk protein loading in dictating functional targeting efficiency.

Together, these results highlight the potential of PNPs as a new class of *protein‐defined biomimetic nanocarriers* capable of mimicking leukocyte‐like vascular interactions with high precision and reproducibility. The ability to engineer BNPs with specific, functionally relevant membrane proteins offers a modular and tunable approach to improve targeting selectivity while reducing off‐target interactions and formulation variability. Beyond inflammation‐targeted delivery, this platform can be readily adapted to incorporate alternative protein repertoires for tissue‐ or disease‐specific applications, paving the way for more rationally designed and clinically translatable biomimetic nanomedicines

## 4 Experimental Section

### 4.1 Materials

Endothelial Cell Medium with supplements from ScienCell. Quick Coating Solution from AngioProteomie. RPMI‐1640, β‐mercaptoethanol, HEPES, sodium pyruvate, glucose, Tween‐20, Phosphate Buffered Saline (PBS), digitonin, triton X‐100, 25 mm Sterile Syringe filters, 0.02 µm PVDF, and cholesterol from Sigma–Aldrich‐Merck. 5X Sample Buffer from A2S. PIPES from Rhenium. 1,2‐dipalmitoyl‐sn‐glycero‐3‐phosphocholine (DPPC), 1,2‐dioleoyl‐sn‐glycero‐3‐phosphocholine (DOPC), and 1,2‐dipalmitoyl‐sn‐glycero‐3‐phosphoethanolamine‐N‐(lissamine rhodamine B sulfonyl) (PE‐Rhod) from Avanti Polar Lipids, Inc. Biotech CE tubing (1000 KDa) from Repligen. Superdex 200 Increase 10/300 GL (Cytiva); NOVA IJM system (HELIX Biotech), Dynamic Light Scattering (DLS), Chemidoc MP Imaging System (Bio‐Rad), Infinite M Plex multimode microplate reader from Tecan. ZetaSizer Nano, and disposable cuvettes for zeta potential measurements from Malvern Instruments. For western blotting Rabbit anti‐CD18 (LS‐C356139‐100) and goat anti‐rabbit IgG‐HRP (ab6721) from Abcam. semi microvolume disposable polystyrene cuvettes for size measurements and MycoStrip Mycoplasma Detection Kit from Tamar Ltd. Trans‐Blot Turbo Mini PVDF membrane, TC20 Automated Cell Counter and slides, and Clarity Western ECL Substrate from Bio‐Rad Laboratories; Pierce BCA kit and Halt protease inhibitor cocktail from Thermo Fisher Scientific, µ‐slide 0.4 was purchased from ibidi.

### 4.2 Cell Lines

THP‐1 human monocytes (American Type Culture Collection (ATCC, TIB‐202), VA, USA) were maintained in RPMI‐1640 complete medium supplemented with 0.05 mm β‐mercaptoethanol, 10 mm HEPES, 1 mm sodium pyruvate, 1% Penicillin/Streptomycin, and a final glucose concentration of 4500 mg/L. HUVEC cells (Lonza, CC‐2519) were cultured in commercially available Endothelial Cell Medium (ECM, ScienCell). Prior to HUVEC seeding, culture flasks were treated with Quick Coat (AngioProteomie) to enhance cell adhesion and growth. Cell cultures were tested for mycoplasma contamination monthly to ensure maintenance of uncontaminated cultures.

### 4.3 Membrane Protein Extraction

MPs were extracted from THP‐1 monocytes using a previously established NEXT protein extraction protocol [33]. Briefly, THP‐1 cells were washed twice with PBS (‐/‐) and centrifuged at 300 g for 10 min at 4°C. The pellet was resuspended in extraction buffer 1 (EB1) at 100 µL per 1 × 10^6^ cells, supplemented with Halt Protease Inhibitor Cocktail (1:100 v/v) and incubated for 10 min at 4°C under gentle agitation, followed by centrifugation at 1,000 × g for 15 min at 4°C. The pellet was resuspended in extraction buffer 2 (EB2) until full pellet solubilization supplemented with Halt Protease Inhibitor Cocktail (1:100 v/v) and then incubated for 30 min at 4°C under gentle agitation, followed by final centrifugation at 5,000 g for 15 min at 4°C. Lastly, the MPs supernatant was aliquoted and stored at −80°C.

### 4.4 Membrane Protein Quantification

The extracted protein concentration was determined using a Pierce BCA Protein Assay kit (Thermo Fisher). A calibration curve using albumin diluted in 1× PBS to the following concentrations was prepared: 0, 25, 125, 250, 500, 750, 1,000, 1,500, and 2,000 µg/mL. For MPs following SEC, a calibration curve using albumin diluted in 1× PBS to the following concentrations was prepared: 0, 25, 50, 100, 200, 300, and 400 µg/mL. MPs were diluted in 1× PBS 1:5 (v/v) and 1:10 (v/v). 20 µL of all samples was loaded in triplicate in a 96‐well microplate and mixed with 200 µL of BCA reagent created by mixing reagents A and B 50:1 (v/v). The plate was incubated at 37°C for 30 min, away from light. The absorbance was measured at 562 nm using a plate reader.

### 4.5 Membrane Protein Size Exclusion Chromatography (SEC)

Membrane protein extracts were separated using size exclusion chromatography (SEC) on an AKTA Avant 25 system equipped with a Superdex 200 Increase 10/300 GL column **(Cytiva)**. The column was equilibrated at room temperature with 300 mm sucrose, 100 mm NaCl, 10 mm PIPES, and 5 mm EDTA, pH 7.4. Samples (up to 500 µL, **∼**8 mg mL^−1^) were loaded onto the column and eluted at 0.75 mL min^−1^, collecting 0.5 mL fractions. Protein elution was monitored by absorbance at 280 nm. Fractions corresponding to the target protein were pooled and stored at −80°C until further use, with 40 µL of each fraction reserved for CD18 western blot analysis.

### 4.6 SDS Gel and Western Blot Detection

Proteins were separated by SDS‐PAGE using 8% gels. Samples were mixed with 5× sample buffer (4:1, v/v), heated at 95°C for 5 min, and 40 µL was loaded per lane. A Blu12 prestained protein ladder was used as a molecular weight marker. Electrophoresis was performed at 80 V for 20 min, followed by 120 V until the dye front reached the bottom of the gel (Bio‐Rad). Gels were rinsed with double‐distilled water and either stained with Coomassie Imperial for total protein visualization or transferred onto PVDF membranes (Trans‐Blot Turbo Transfer Pack, 0.2 µm) using the Trans‐Blot Turbo Transfer System (Bio‐Rad).

Membranes were blocked with 5% BSA in PBST (0.05% Tween‐20 in PBS) for 1 h at room temperature and then incubated overnight at 4°C with a primary anti‐human CD18 antibody (Abcam, ab307406; 1:1000 in 2% BSA in PBST). After washing with PBST, membranes were incubated with an HRP‐conjugated secondary antibody (Goat Anti‐Rabbit IgG H&L, Abcam, ab6721; 1:10,000) for 1 h at room temperature. Protein bands were visualized by HRP chemiluminescence and imaged using a ChemiDoc imaging system (Bio‐Rad), exposure time 0.075 s.

### 4.7 PNPs Synthesis

NPs were prepared by microfluidic mixing using a Helix instrument equipped with an IJM (Impinged Jet Mixer, size O), which enables rapid and efficient microscale mixing. Lipids (DPPC:DOPC:Cholesterol, 4:3:3 molar ratio) were dissolved in ethanol at 10 mm, heated to 45°C, and sonicated for 1 min prior to mixing. For fluorescently labeled NPs, Liss Rhod‐PE (16:0) was added at 50 µL of 1 mg mL^−1^ per 1 mL of NPs. Lipid solutions were mixed with a 37°C PBS aqueous phase containing THP‐1–derived MPs at an organic:aqueous flow rate ratio (FRR) of 1:5. The total flow rate (TFR) was 2.2 mL·min^−1^ for liposomes, Leukosomes, and PNPs, and 2.4 mL·min^−1^ for high‐Leukosomes. MPs were incorporated at protein:lipid ratios of 1:100 for Leukosomes and PNPs, and 1:20 for high‐Leukosomes. Following mixing, solvents and non‐incorporated proteins were removed by dialysis using 1000 kDa membrane tubing (Biotech CE Tubing, Repligen), and particles were sterile‐filtered (0.2 µm) and stored at 4°C until use.

### 4.8 PNPs Characterization for Physicochemical Properties: Size, PDI, ZP, and NPs Concentration and Stability Test

The hydrodynamic size, polydispersity index (PDI), ζ‐potential, and concentration of NPs were measured by dynamic light scattering (DLS) using a Zetasizer Ultra (Malvern Panalytical) at 25°C. Samples were diluted 1:100 in PBS for size, PDI, and concentration measurements, and in 10% PBS in DDW for ζ‐potential. For rhodamine‐labeled NPs, a fluorescent filter was included during measurements. Colloidal stability was assessed by monitoring size, PDI, and ζ‐potential over time under storage at 4°C on days 1, 4, and 14. All measurements were performed in triplicate on independently prepared NP batches, and results are reported as mean ± standard deviation (SD).

### 4.9 Cryo‐TEM Imaging

NPs were diluted to ∼3 mm in PBS. Then, a small drop, ca. 3 µL, was applied to a perforated carbon film supported on a standard TEM grid (Ted Pella). The drop was then blotted into a thin liquid film spanning the holes by bringing it into contact with a piece of filter paper. This entire process was performed in the Leica GP2 plunge freezing chamber at ambient temperature, saturated with water vapors [38]. The specimen was plunged into freezing ethane, at −180°C and loaded into a TEM cooling holder using a dedicated transfer station. During imaging, the holder tip was kept at about−180°C to avoid sublimation and to preserve the supercooled state of the vitreous ice. The imaging was performed with minimum electron dose possible (about 10 e–/Å2) on Talos F200X FEG TEM (Thermo Fisher) using a Volta phase plate for contrast enhancement [38]. The micrographs were recorded with a direct Falcon electron camera through TIA software.

### 4.10 Proteomic Sample Preparation and Mass Spectrometry Analysis

Proteins integrated within the NPs were isolated by precipitation with chilled acetone (final concentration 80%) and incubated overnight at –20°C. The resulting pellets were washed three times with cold acetone, air‐dried, and solubilized in 8.5 m urea containing 100 mm ammonium bicarbonate and 10 mm dithiothreitol (DTT). Protein content was determined using the Bradford assay. Samples were then reduced at 60°C for 30 min, alkylated with 35.2 mM iodoacetamide for 30 min at room temperature in the dark, and digested with sequencing‐grade trypsin at an enzyme‐to‐substrate ratio of 1:50 (w/w) overnight at 37°C, followed by a second digestion step for 4 h using a 1:100 ratio. Peptide mixtures were purified with Oasis HLB µElution plates (Waters), vacuum‐dried, and reconstituted in 0.1% formic acid prior to LC‐MS/MS analysis. Peptides were separated on a 30 cm C18 analytical column (75 µm ID) using an Ultimate 3000 UHPLC system coupled to a Q‐Exactive HFX mass spectrometer (Thermo Fisher Scientific). A linear gradient of 5–28% acetonitrile in 0.1% formic acid was applied over 120 min, followed by a 15‐min increase to 95% acetonitrile. The instrument operated in positive ion mode over an m/z range of 350–1200, with MS1 and MS2 resolutions set to 120 000 and 20 000, respectively. Higher‐energy collisional dissociation (HCD) was used for fragmentation of the top 20 most intense precursor ions per cycle.

Raw mass spectrometry files were processed in MaxQuant (version 2.4.2.0) [39]. Peptide identification was performed with the Andromeda search engine using trypsin as the specific protease, permitting up to two missed cleavages. Carbamidomethylation of cysteine residues was defined as a fixed modification, while methionine oxidation and N‐terminal acetylation were considered variable modifications. Data were searched against the Homo sapiens reference proteome (UniProt, August 2023; 20 424 entries) using a target‐decoy approach. Peptide‐spectrum matches (PSMs), peptides, and proteins were filtered at a 1% false discovery rate (FDR). Peptides shorter than seven amino acids were excluded, and quantification was based on razor and unique peptides. The “match between runs” function was activated with a retention‐time alignment window of 1 min to improve peptide transfer across samples. Reverse hits and known contaminants were omitted from downstream analysis.

Quantitative and statistical analyses were performed using Perseus (version 2.0.10.0). LFQ (label‐free quantification) intensity values were log_2_‐transformed to normalize protein abundance and enable quantitative comparison across samples. Only proteins with valid measurements in all three replicates of at least one group were included. Differential protein expression was assessed using a two‐sample t‐test (FDR = 0.05, |log_2_ fold change| ≥ 1, *p* ≤ 0.05). Heatmaps of adhesion‐related MPs present log_2_‐transformed LFQ intensities (*n* = 3).

### 4.11 LPS and PNPs Toxicity

HUVECs were seeded in Quick Coat–coated 96‐well plates at 8,000 cells/well and allowed to adhere for 24 h in complete endothelial growth media at 37°C and 5% CO_2_. Cells were treated with 100 ng/mL LPS (Escherichia coli O26:B6, Sigma–Aldrich, L8274) for 24 h, followed by incubation with 0.1 mM nanoparticles (without dye) for 1 h [32]. Cell viability was assessed using the MTT assay by adding 0.5 mg/mL MTT in complete media for 2 h at 37°C. Formazan crystals were dissolved in DMSO, and absorbance was measured at 540 nm. Viability was expressed relative to untreated controls, and all measurements were performed in triplicate on independently prepared NP batches.

### 4.12 Static PNPs Adhesion Evaluation by Confocal and Cytation Imaging

HUVECs were seeded in Quick Coat–coated 96‐well plates at 8,000 cells/well and allowed to adhere for 24 h in complete endothelial growth media at 37°C and 5% CO_2_. Cells were stimulated with LPS at 100 ng/mL for 24 h. After stimulation, cells were washed with PBS (+/+) and incubated with rhodamine‐labeled nanoparticles (0.1 mm) in complete media for 1 h. Following incubation, cells were washed with PBS (+/+), fixed with 4% paraformaldehyde for 10 min, and stained with Hoechst (Thermo Fisher; 1:10,000, 10 min, 37°C) to label nuclei. Images for detailed analysis were acquired using a spinning disk confocal microscope (SDC, Nikon, LS&E Infrastructure Center, Technion). For high‐throughput screening, imaging was performed using a Cytation 5 plate reader (BioTek). Data were analyzed to assess nanoparticle adhesion and uptake. Experiments were performed in triplicate using independently prepared biological replicate batches. The fluorescence of rhodamine‐labeled nanoparticles (NPs) was measured for each batch and normalized to the relative fluorescence of the NPs to enable comparison across experiments. Image analysis was performed using FIJI (ImageJ) software.

### 4.13 PNPs Adhesion at Static Conditions Using Flow Cytometry

HUVECs were seeded in Quick Coat–coated 6‐well plates at 500,000 cells/well and allowed to adhere overnight in complete endothelial growth media at 37°C and 5% CO_2_. Cells were stimulated with LPS (100 ng/mL) for 24 h. After stimulation, cells were washed with PBS (+/+) and incubated with rhodamine‐labeled nanoparticles at 1, 0.1, or 0.01 mM in complete media for 15 min or 1 h. Following incubation, cells were washed twice with PBS (+/+), detached using trypsin, and resuspended in ECM. Samples were analyzed immediately using a spectral flow cytometer (Aurora, Cytek). Data were processed to determine the percentage of NP‐positive cells and mean fluorescence intensity. The fluorescence of rhodamine‐labeled nanoparticles (NPs) was measured for each batch and normalized to the relative fluorescence of the NPs to enable comparison across experiments.

### 4.14 PNPs Adhesion Under Flow

HUVECs were cultured to confluence in an ibidi µ‐Slide 0.4 Luer (ibidi) and activated with LPS (100 ng mL^−1^, 24 h, 37°C). Rhodamine‐labeled PNPs (0.05 mM in PBS (+/+)) were equilibrated to 37°C prior to perfusion. NPs suspensions were flowed through the channel using a syringe pump (BS‐300, Braintree Scientific) at 0.57 ± 0.02 mL min^−1^ for 12 min.

Shear stress was calculated based on the experimental setup using PBS (+/+) (pH 7.4) at 37°C in a µ‐Slide 0.4 Luer (ibidi). To achieve shear stress of approximately 0.5 dyne cm^−^^2^, the corresponding flow rate was determined according to the manufacturer's formulation [40] which relates shear stress, channel geometry, and fluid viscosity. Based on these parameters, a flow rate of 0.5 mL min^−1^ was selected for all experiments.

(1) $$\Phi =\frac{\tau }{\eta \;X}\;$$

here, Φ is the flow rate [mL min ^−1^], τ is the shear stress [dyn cm^−2^], η is the dynamic viscosity, and X presents the channel‐specific geometry factor (X = 131.6 for the µ‐Slide 0.4 Luer). Following perfusion, channels were gently washed with PBS at 0.2 mL min^−1^ for 30 s to remove the residual nanoparticle suspension, ensuring that only nanoparticles accumulated on the endothelium were analyzed. Fluorescence imaging was performed immediately after using identical acquisition settings across all samples. The fluorescence of rhodamine‐labeled NPs was measured for each batch and normalized to the relative fluorescence of the NPs to enable comparison across experiments. Image analysis was performed using FIJI (ImageJ) software.

### 4.15 Artificial Intelligence (AI) and AI‐Assisted Technologies

While preparing this article, AI‐assisted technologies, including ChatGPT, were utilized to improve its readability and language. Afterward, we meticulously reviewed and edited the material as required, taking full responsibility for the content of this document.

## Author Contributions

A.Z. conceived this research. A.Z. and S.A.R. designed the experiments, processed, and analyzed the data. S.A.R. and M.B.E. cultured the cells and extracted MPs. S.A.R. separated MPs using the ÄKTA system and characterized protein enrichment. S.A.R. synthesized the nanoparticles (NPs). S.A.R. and M.B.E. characterized the NPs. S.A.R. and R.M. analyzed the proteomics data. S.A.R. performed the in vitro toxicity, accumulation, and imaging experiments, as well as image analysis. T.G.L. provided guidance on proteomics and image analysis. S.A.R. and A.Z. wrote and edited the manuscript.

## Conflicts of Interest

The authors declare no conflicts of interest.

## Supporting information




**Supporting File**: smll72206‐sup‐0001‐SuppMat.docx

## Data Availability

The data that support the findings of this study are available in the supplementary material of this article.
