# Supplementary material for: Selective Membrane Protein Enrichment Enables Defined Biomimetic Nanoparticles for Endothelial Targeting
Source: Small. 2026 Jan 12;22(11):e13548. doi: 10.1002/smll.202513548 (PMC12921465; doi:10.1002/smll.202513548)
Supplement: Supplementary file 1 — Supporting File: smll72206‐sup‐0001‐SuppMat.docx [file SMLL-22-e13548-s001.docx]

Supporting Information


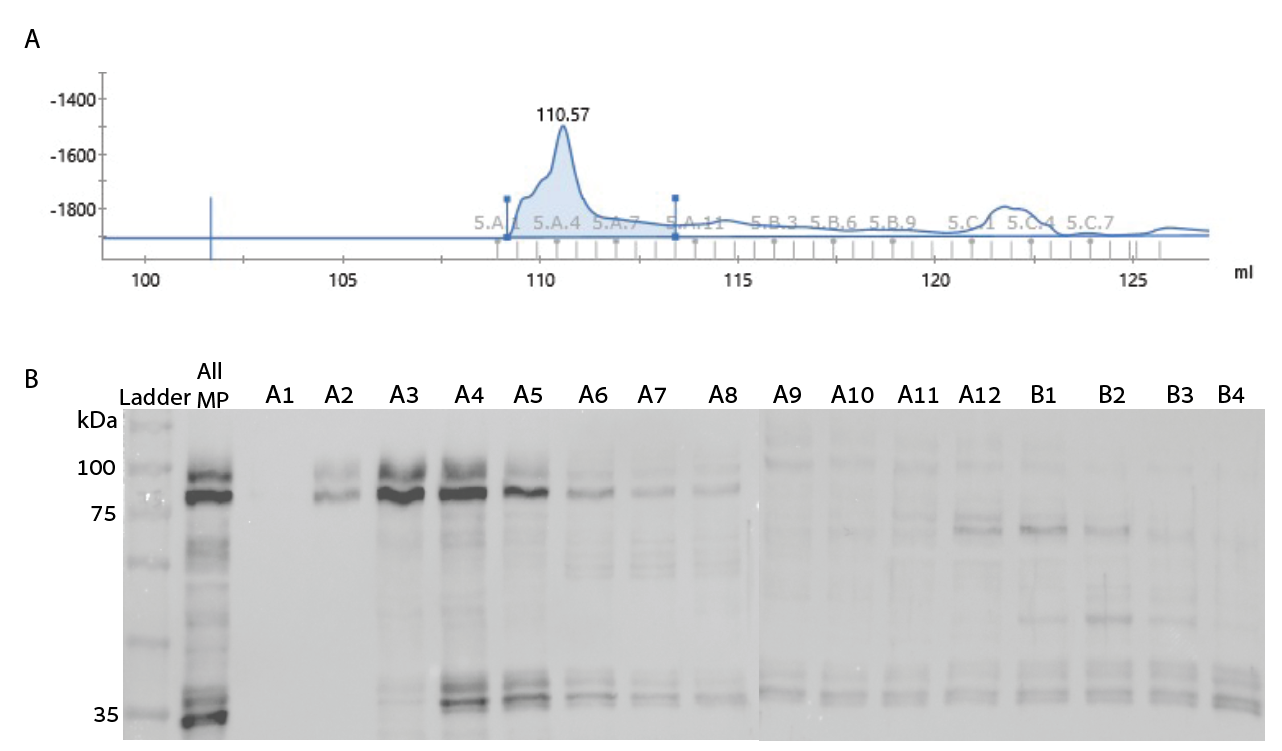


**Supplementary 1. Adhesion MPs enrichment process (A) SEC profile monitored at 280 nm using AKTA. (B) CD18 Western blot.**


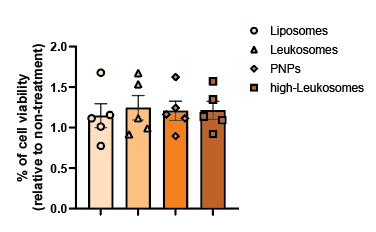


**Supplementary 2. Cytotoxicity of 0.1 mM BNPs on healthy HUVECs after 1 h incubation. Data are presented as mean ± SEM (n = 5). Statistical analyses was performed using one‐way ANOVA followed by Tukey’s multiple comparisons test (**P*<0.05).**

**Table Supplementary 1: Effect of Triton X-100 concentration on BNPs stability.**

|  | TFR  [mL min^-1^] | % triton in the buffer | V Buffer per 1mL NPs | Triton Final [%] | Z average [nm] | conclusion |
| --- | --- | --- | --- | --- | --- | --- |
| PNPs | 2.5 |  | 0.097 |  | 355 | Final Triton X-100 concentration was too high |
|  | 2.5 |  | 0.097 |  | 375 |  |
|  | 2.5 |  | 0.097 |  | 406 |  |
|  | 2.5 |  | 0.083 |  | 380 |  |
|  | 4 |  | 0.063 |  | 500 |  |
|  | 4 |  | 0.058 |  | 400 |  |
| Leukosomes | 2.5 | 0.5 | 0.083 | 0.042 | 104 | C < 0.1% |
| Liposomes | 2.5 | 0.5 | 0.083 | 0.042 | 114 | C < 0.1% |
|  | 2.5 | 1.0 | 0.125 | 0.125 | **400** | C > 0.1%, |
|  | 2.5 | 1.5 | 0.054 | 0.081 | 146 | C < 0.1% |
|  | 3.5 | 1.5 | 0.054 | 0.081 | 120 | Increased TFR results in reduced size |
|  | 2.5 | 1.5 | 0.058 | 0.086 | 120 | C < 0.1% |
|  | 4 | 1.5 | 0.080 | 0.120 | **285** | higher TFR did not yield ~100nm size at C > 0.1% |

**Abbreviations**

BNPs: biomimetic nanoparticles

DLS: dynamic light scattering

EB1 and EB2: extraction buffer 1 and 2

ECM: endothelial cell medium

HUVEC: human umbilical vein endothelial cells

LPS: lipopolysaccharide

MPs: membrane proteins

NPs: nanoparticles

PDI: polydispersity index

PNPs: particular nanoparticles (or platform nanoparticles)

SDC: spinning disc confocal

SEC: size exclusion chromatography

VCAM-1: vascular cell adhesion molecule 1

WB: western blot

WSS: wall shear stress
